# Case report: maternal mosaicism resulting in inheritance of a novel *GATA6* mutation causing pancreatic agenesis and neonatal diabetes mellitus

**DOI:** 10.1186/s13000-016-0592-1

**Published:** 2017-01-03

**Authors:** Daphne Yau, Elisa De Franco, Sarah E. Flanagan, Sian Ellard, Miriam Blumenkrantz, John J. Mitchell

**Affiliations:** 1 Division of Pediatric Endocrinology Montreal Children’s Hospital, McGill University Health Centre, 1001 Boulevard Decarie, Montreal, H4A 3J1 Quebec Canada; 2 Institute of Biomedical and Clinical Science, University of Exeter Medical School, Barrack Road, Exeter, EX2 5DW UK; 3 Department of Pathology Montreal Children’s Hospital, McGill University Health Centre, 1001 Boulevard Decarie, Montreal, H4A 3J1 Quebec Canada

**Keywords:** GATA6, Haploinsufficiency, Mosaicism, Pancreas abnormalities, Neonatal diabetes mellitus

## Abstract

**Background:**

Haploinsufficiency of the *GATA6* transcription factor gene was recently found to be the most common cause of pancreatic agenesis, a rare cause of neonatal diabetes mellitus. Although most cases are *de novo*, we describe three siblings with inherited *GATA6* haploinsufficiency and the rare finding of parental mosaicism.

**Case Presentation:**

The proband was born at term with severe intrauterine growth restriction, the first child of non-consanguineous parents. Diabetes occurred on day of life 1 with pancreatic exocrine insufficiency noted at several months of age. Pancreatic agenesis with absent gallbladder was confirmed when he underwent congenital diaphragmatic hernia and intestinal malrotation repair. A patent ductus arteriosus and pulmonary stenosis were repaired in infancy. Neurocognitive development has been normal. A second pregnancy was terminated due to tetralogy of Fallot and pulmonary hypoplasia secondary to congenital diaphragmatic hernia. The fetus also demonstrated severe pancreatic hypoplasia, gallbladder agenesis and intestinal rotation abnormalities. Despite severe hypoplasia, the pancreas demonstrated normal islet histology. Another sibling was found to have multiple cardiac abnormalities, requiring procedural intervention. Given the proband’s spectrum of congenital anomalies, Sanger sequencing of the *GATA6* gene was performed, revealing a novel heterozygous c.635_660del frameshift mutation (p.Pro212fs). The mutation is predicted to be pathogenic, resulting in inclusion of a premature stop codon and likely degradation of the gene transcript by nonsense-mediated decay. The abortus and the sibling with the cardiac defect were both found to have the mutation, while the father and remaining sibling were negative. The mother, who is healthy with no evidence of diabetes or cardiac disease, is mosaic for the mutation at a level of 11% in her peripheral leukocytes by next-generation sequencing.

**Conclusion:**

We highlight a rare mechanism of pancreatic agenesis, this being only the second report of parental mosaicism for a *GATA6* mutation and one of a handful of inherited cases. We also further define the phenotypic variability of *GATA6* haploinsufficiency, even in individuals carrying the same mutation. Mutations in *GATA6* should be strongly considered in cases of diabetes due to pancreatic hypoplasia or agenesis, and potentially affected family members should be tested regardless of phenotype.

## Background

Although type 1 diabetes mellitus accounts for the vast majority of pediatric diabetes, monogenic forms account for up to 4% of cases [1, 2]. The latter are due to single gene defects affecting pancreatic β-cell function, development or survival and include neonatal diabetes mellitus (NDM). NDM is defined as diabetes occurring in the first six months of life and occurs with an estimated frequency of approximately 1:100 000 live births [2–5]. Both transient and permanent forms of NDM exist (Table 1). Although clinically indistinguishable from each other at presentation, most transient cases resolve at a median age of 12 weeks but relapse in 50–60% [6, 7]. Different genetic loci and genes are associated with transient versus permanent NDM. Methylation abnormalities at chromosome 6q24 account for two thirds of transient cases, while in permanent NDM, activating mutations in *ABCC8* and *KCNJ11* are the most common genetic defects in outbred populations and *EIF2AK3* is the most frequent in consanguineous groups [3, 7]. *KCNJ11* and *ABCC8* encode the KIR6.2 and SUR1 subunits of the K_ATP_ channel, respectively, which links glucose metabolism to insulin secretion. *EIF2AK3* encodes a kinase involved in regulating the endoplasmic reticulum stress response to misfolded proteins [8, 9]. Although the mechanism causing diabetes is unclear, defects in *EIF2AK3* may cause ER stress in the β-cell from misfolded proteins due to the high demand for insulin secretion, eventually leading to β-cell apoptosis. Alternatively, diabetes may be linked to reduced β-cell proliferation with abnormal insulin trafficking and secretion, as observed in *EIF2AK3* knockout mice [9].

**Table 1:** Key genes associated with neonatal diabetes mellitus

Gene	Location	Inheritance	Clinical features
*PLAGL1, HYMAI*	6q24	Variable (imprinting)	Transient NDM
			± Macroglossia, ± Umbilical Hernia, ± Other features if part of generalized hypomethylation syndrome
*KCNJ11*	11p15.1	De novo, Dominant or Recessive	Permanent or Transient NDM, ± Developmental delay, epilepsy, neonatal diabetes (DEND) syndrome
*ABCC8*	11p15.1	De novo, Dominant or Recessive	Transient or Permanent NDM, ± DEND syndrome
*EIF2AK3*	6q22.1	Recessive	Wolcott-Rallison Syndrome: Permanent NDM Short stature Bone dysplasia Hepatic dysfunction
*INS*	11p15.5	Recessive	Isolated permanent or transient NDM
*GCK*	7p15-p13	Recessive	Isolated permanent NDM

The most common genes associated with neonatal diabetes mellitus are described with key clinical features and mode of inheritance. Adapted from [2]. Genes associated with abnormal pancreas development are described in Table 2

Permanent NDM can also result from pancreatic agenesis or hypoplasia, which has been linked to mutations in transcription factors important for pancreatic and β-cell development. The most common of these is *GATA6* [10]. Other causes include mutations in *PDX1* and the *PTF1A* enhancer causing isolated abnormal pancreas development, while mutations in *PTF1A*, *RFX6*, *HNF1B* and *GATA4* are associated with both pancreatic and extra-pancreatic abnormalities (Table 2) [11–16]. *GATA6* is one of a family of evolutionarily conserved transcription factors recognizing the A/T-GATA-A/G consensus sequence, with key roles in the development and differentiation of multiple cell lineages and tissues [17, 18]. Heterozygous inactivating mutations in *GATA6* were identified through a whole exome sequencing strategy in 15/27 (56%) individuals with pancreatic agenesis, defined as NDM requiring insulin treatment and exocrine pancreatic insufficiency requiring enzyme replacement. This is a syndromic form of NDM associated with extra-pancreatic features including cardiac, hepatobiliary, gastrointestinal, neurocognitive and other endocrine involvement. Since its initial discovery, the phenotypic spectrum of *GATA6* haploinsufficiency in humans has proven to be more diverse than initially appreciated, and although the initial cases were *de novo*, several instances of inherited *GATA6* mutations have now been described [19–21]. This report describes three siblings with inherited *GATA6* haploinsufficiency and the rare finding of parental mosaicism.

**Table 2:** Genes associated with neonatal diabetes mellitus and abnormal pancreas development

Gene	Location	Inheritance	Clinical features	Reference
*PDX1*	13q12.1	Recessive	IUGR, Pancreatic agenesis, Permanent NDM, PI	[11]
*PTF1A* enhancer	10p12.2	Recessive	IUGR, Pancreatic agenesis, Permanent NDM, PI	[12]
*PTF1A*	10p12.2	Recessive	IUGR, Pancreatic agenesis, Permanent NDM, variable PI, Cerebellar hypoplasia/aplasia	[13]
*RFX6*	6q22.1	Recessive	IUGR, Annular/hypoplastic pancreas, Permanent NDM, Chronic diarrhea, Biliary and intestinal abnormalities	[14, 32–35]
*HNF1B*	17q21.3	Dominant	IUGR, Pancreas hypoplasia, Renal cysts	[15]
*GATA4*	8p23.1	Dominant	IUGR, Pancreatic hypoplasia/agenesis, Congenital heart defects, Developmental delay	[16]

Genes in addition to GATA6 associated with both isolated diabetes and extra-pancreatic features are described with key clinical features and mode of inheritance

## Case presentation

The proband was born at 37 weeks gestation after a pregnancy complicated by severe intrauterine growth restriction, reflected in the birth weight of 1.74 kg (<3rd percentile, −4 standard deviations). He was the first child of non-consanguineous parents of French Canadian background. Hyperglycemia occurred within the first 12 h of life and neonatal diabetes mellitus was diagnosed. An initial ultrasound visualized only the head of the pancreas, suggesting pancreatic hypoplasia. He was placed on subcutaneous insulin therapy with adequate glycemic control. Moderate valvular pulmonary stenosis and a patent ductus arteriosus (PDA) were also diagnosed and valvuloplasty was performed in the neonatal period.

At several months of age, recurrent episodes of hypoglycemia with decreasing insulin requirements began to occur. He was diagnosed with pancreatic exocrine insufficiency (PI) based on the recurrent hypoglycemia, poor weight gain and low stool fecal elastase, leading to initiation of pancreatic enzyme replacement. At 8 months of age, he was readmitted for severe failure to thrive and hypoglycemia secondary to inadequately treated PI, heart failure from the persistent PDA and a previously undiagnosed congenital diaphragmatic hernia. Intestinal malrotation was also noted. His medical management was optimized and he underwent repair of the PDA, diaphragmatic hernia and intestinal malrotation. At the time of surgery, no pancreatic tissue or gallbladder could be identified, demonstrating both pancreatic and gallbladder agenesis.

Despite improvement in his nutritional and overall health status, he continued to have episodes of early morning hypoglycemia, presumably due to deficient glucagon secretion. This was managed with feeds of uncooked cornstarch to provide a source of slowly-released glucose while on insulin injections and, when transitioned to pump therapy, with low basal insulin rates. Control of both the diabetes and exocrine insufficiency have been adequate with no evidence of malabsorption. There has been no clinical evidence of other endocrine dysfunction and thyroid function testing has been normal. He has also demonstrated appropriate neurocognitive development as of 9 years of age. Previous testing of the *PDX1* gene, a recognized cause of pancreatic agenesis (Table 2) as well as broader genetic analysis through the 1,000,000 Single Nucleotide Polymorphism project were unrevealing.

**Fig. 1:**
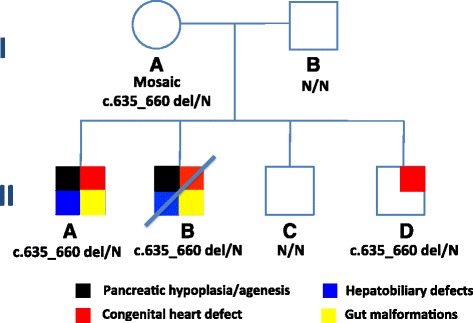
Family Pedigree. The proband (II-A) and abortus (II-B) have multiple congenital anomalies including severe pancreatic agenesis/hypoplasia, while one sibling (II-D), also heterozygous for the same mutation, has isolated congenital cardiac anomalies including a PDA, left pulmonary artery stenosis, moderate atrial septal defect. The father and remaining sibling are negative for the mutation. The genotypes are provided below each symbol, N denotes the wildtype allele

A second pregnancy was terminated at 20 weeks due to complex congenital heart disease. Examination of the abortus revealed a male fetus with tetralogy of Fallot, congenital diaphragmatic hernia, severe pancreatic hypoplasia, gallbladder agenesis and intestinal non-rotation (Fig. 1, individual IIB). Subsequently, two male infants were born. The youngest child was found to have a PDA requiring procedural intervention, left pulmonary artery stenosis, and moderate atrial septal defect (Fig. 1, individual IID). Neither the remaining sibling nor either parent have any clinical evidence of diabetes, pancreatic exocrine insufficiency or cardiac disease.

## Methods

### GATA6 sequencing

Genomic DNA was extracted from peripheral leukocytes using standard procedures. Exons 2–7 and the exon/intron boundaries of the *GATA6* gene were analyzed by Sanger sequencing as described previously. Exon 1 is a non-coding exon and was not sequenced [10]. Sequencing reactions were run on an ABI3730 capillary machine (Applied Biosystems, Warrington, U.K.) and analyzed using Mutation Surveyor v4.0.6 (SoftGenetics, State College, PA) (*GATA6* nucleotide reference NM_005257.3). Allele frequency was quantified by next-generation sequencing as previously described [22].

### Histology

Immunohistochemistry and staining for hematoxylin, phloxine, saffron were performed using standard procedures on slides of pancreatic tissue obtained at autopsy from the abortus (Fig. 1, individual IIB). For the immunohistochemistry, antibodies were used to detect insulin, glucagon and somatostatin.

## Results

**Fig. 2:**
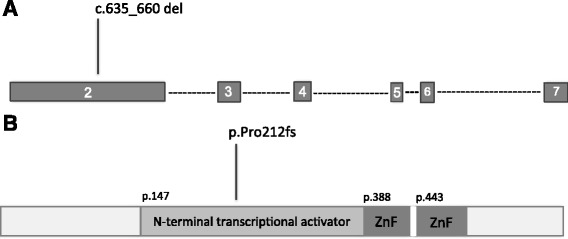
Genomic and protein positions of the novel *GATA6* mutation. **a** Exons 2 to 7 with the location of the novel deletion described are shown. **b** The frameshift resulting from the deletion is shown with its location in the transcriptional activation domain. The numbers refer to the amino acid position of the latter domain and the DNA-binding zinc fingers (ZnF)

Given the similarity between the proband’s spectrum of congenital anomalies and the clinical phenotype of *GATA6* mutations, Sanger sequencing of exons 2–7 of the *GATA6* gene was performed in peripheral leukocytes. A novel c.635_660del frameshift mutation (p.Pro212fs) was identified in the proband. The mutation is located in exon 2, within the transcriptional activation domain, and is predicted to result in inclusion of a premature stop codon and likely degradation of the gene transcript by nonsense-mediated decay (Fig. 2). The mother, the abortus and the sibling with the congenital heart defect were also found to carry the mutation (Fig. 1). Notably, the mother, who has no clinical evidence of diabetes, including gestational diabetes or cardiac disease, was found to be mosaic for the mutation (Fig. 1, individual IA). This was confirmed and quantified by next-generation sequencing, revealing 11% mosaicism in peripheral blood. The father and the remaining sibling’s testing were negative.

**Fig. 3:**
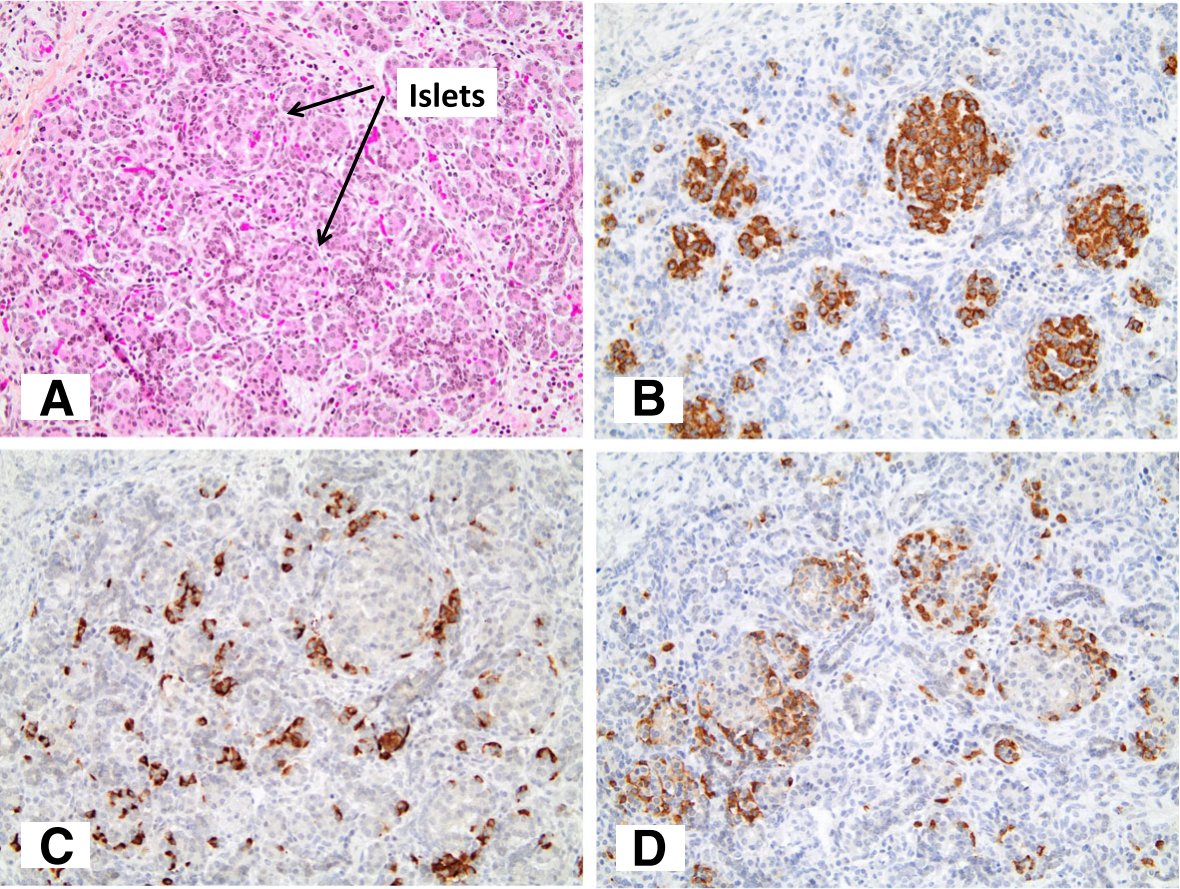
Islet morphology is preserved with appropriate distribution of insulin- and glucagon-positive cells despite severe pancreas hypoplasia. Pancreas sections from individual II-B in Fig. 1 were stained using haematoxylin, phloxine and saffron staining (**a**), and immunohistochemistry for insulin, glucagon and somatostatin (**b** to **d**). Islets were identified (**a**) demonstrating normal morphology with central insulin (**b**), peripheral glucagon (**c**) and somatostatin staining (**d**). All images were photographed at 200× magnification

Histological analysis of the pancreas from the abortus (Fig. 1, individual IIB) demonstrated well-formed islets despite severe pancreatic hypoplasia (Fig. 3). The majority of islet cells stained positive for insulin and were surrounded by smaller populations of glucagon and somatostatin positive cells, demonstrating normal islet morphology.

## Discussion

We describe three siblings heterozygous for the same *GATA6* mutation inherited via parental mosaicism, yet with widely different manifestations, further defining the clinical phenotype associated with this rare disease. Since the initial report of heterozygous *GATA6* mutations as the most common cause of pancreatic agenesis or hypoplasia, a much greater degree of phenotypic variability has been recognized for both pancreatic and extra-pancreatic features [10, 19]. Diabetes mellitus is the most common pancreatic feature in 98% of cases (Table 3). Although the majority of cases present with NDM, in others diabetes develops later in life or has yet to occur by adulthood. Similarly, although clinical PI has been reported in 81% of cases, subclinical PI and normal exocrine function have also been described (Table 3) [10, 19, 23]. Of the extra-pancreatic features, cardiac defects are the most common, in 88% of cases, although gastrointestinal, hepatobiliary, neurodevelopmental and other endocrine involvement have also been reported (Table 3) [10, 19–21, 23–29]. The family described here illustrates this clinical variability. The proband has NDM and PI, with significant cardiothoracic, gastrointestinal and biliary involvement. A similar phenotype was seen in the second affected sibling whereas in contrast, the third sibling has isolated cardiac involvement. Thus, *GATA6* haploinsufficiency needs to be strongly considered as a potential cause in cases of diabetes secondary to abnormal pancreas development and, if confirmed, a thorough, multi-system assessment, particularly cardiac, should be performed to evaluate for other abnormalities. Consideration should also be given to screening for glycemic abnormalities in those whom diabetes has not yet developed.

**Table 3:** Phenotypic spectrum associated with *GATA6* haploinsufficiency

Clinical features	Spectrum and severity	Patients with GATA6 mutation, *N* = 41 (%)
Pancreatic Features		
Diabetes Mellitus	Neonatal/adult-onset	Total 40/41 (98), NDM 33/41 (81)^a^, Child-onset 4/41 (10)^b^, Adult-onset 3/41 (7)
Exocrine Insufficiency	Clinical/subclinical^d^	Total 33/37 (89)^c^, Clinical 30/37 (81)
Extra-Pancreatic Features		
Cardiac	Isolated septal defect/multiple complex defects	36/41 (88)
Hepatobiliary	Gallbladder atresia, biliary atresia	13/41 (32)
Gastrointestinal	Umbilical hernia, diaphragmatic hernia, intestinal malrotation	8/41 (20)
Other Endocrine	Hypothyroidism, pituitary agenesis	7/41 (17)
Neurocognitive	Developmentally appropriate/mild learning difficulties/severe developmental delay	13/41 (32)
Genitourinary	Bicornuate uterus, hydronephrosis, hydroureter	2/41 (5)

Summary of the pancreatic and extra-pancreatic features of the published cases to date, demonstrating the variability of the phenotype [10, 19–21, 23–29]. ^a^NDM group includes one case of transient NDM, ^b^childhood-onset group includes one case of impaired glucose tolerance in adolescence, ^c^four cases were excluded as no information on exocrine insufficiency was provided, ^d^subclinical was defined as low fecal elastase or positive fecal fat in the absence of clinical symptoms or need for pancreatic enzyme replacement

Although most cases of *GATA6* mutations are *de novo*, several cases of dominant inheritance have been reported [10, 19]. The family described here is notable for being amongst these inherited cases and moreover, is only the second report of parental mosaicism [19]. In the previous report, the parent was known to have a congenital heart defect, whereas in our case the mother has no known cardiac abnormalities or pancreatic phenotype. Presumably differing levels of tissue mosaicism is key. Since three out of four offspring inherited the mutation, the level of mosaicism is likely significantly higher in the germline compared to other tissues in our case given the absence of other system involvement and almost certainly higher than the 11% detected in peripheral blood. Accordingly, testing of parents and other potentially affected family members should be performed, even in individuals without an obvious clinical phenotype.

The mechanism underlying the variable expressivity of *GATA6* haploinsufficiency remains unclear with no obvious genotype-phenotype relationship [19]. Histologically, grossly preserved islet morphology was observed, which contrasts with murine models demonstrating abnormal morphogenesis with failure of progenitor cells to differentiate into endocrine and exocrine lineages (Fig. 3). However, humans appear to have greater sensitivity to *GATA6* gene dosage as both *Gata4* alleles in addition to at least one *Gata6* allele must be inactivated in mice to cause pancreatic abnormalities [30, 31]. Although not explored in this report, at a molecular level, there are several potential mechanisms. *In vitro* studies have shown that missense mutations affecting highly conserved zinc finger residues result in failure to bind target promoters in luciferase reporter promoter assays [10, 19, 27]. Given the location of the novel frameshift mutation described here in the transactivation domain, there could be an impact on downstream genetic targets through altered binding of co-regulatory factors. However, the most likely explanation is that the introduction of a premature termination codon results in a reduction in *GATA6* mRNA transcript levels through nonsense-mediated decay, as previously described [26]. Variable efficiency of this decay in different tissues could contribute to the phenotypic variability. Modifier genes, epigenetic mechanisms and environmental factors represent other potential mechanisms that could also contribute to the variability associated with *GATA6* heterozygous mutations.

## Conclusions

Heterozygous mutations in the *GATA6* transcription factor gene are the most common cause of neonatal diabetes due to pancreatic agenesis. We describe three siblings heterozygous for the same *GATA6* mutation inherited via parental mosaicism, a rare occurrence. Their markedly different manifestations illustrate both the importance of *GATA6* for pancreas development as well as the phenotypic variability of *GATA6* haploinsufficiency. Elucidating the mechanisms underlying the phenotype and its variability will improve our understanding of pancreas development and may provide insights into the mechanisms underlying other forms of abnormal pancreas development and neonatal diabetes.

## Abbreviations

IUGR: intrauterine growth restriction
NDM: neonatal diabetes mellitus
PDA: patent ductus arteriosus
PI: pancreatic exocrine insufficiency
